# Privacy-preserving for assembly deviation prediction in a machine learning model of hydraulic equipment under value chain collaboration

**DOI:** 10.1038/s41598-022-14835-1

**Published:** 2022-06-24

**Authors:** Hao Qiu, Yixiong Feng, Zhaoxi Hong, Kangjie Li, Jianrong Tan

**Affiliations:** 1 State Key Laboratory of Fluid Power and Mechatronic Systems, Zhejiang University, Hangzhou, 310027 People’s Republic of China; 2 Engineering Research Center for Design Engineering and Digital Twin of Zhejiang Province, Zhejiang University, Hangzhou, 310027 People’s Republic of China; 3 Department of Mechanical and Aerospace Engineering, The Hong Kong University of Science and Technology Clear Water Bay, Kowloon, Hong Kong

**Keywords:** Mechanical engineering, Computer science

## Abstract

Hydraulic equipment, as a typical mechanical product, has been wildly used in various fields. Accurate acquisition and secure transmission of assembly deviation data are the most critical issues for hydraulic equipment manufacturer in the PLM-oriented value chain collaboration. Existing deviation prediction methods are mainly used for assembly quality control, which concentrate in the product design and assembly stage. However, the actual assembly deviations generated in the service stage can be used to guide the equipment maintenance and tolerance design. In this paper, a high-fidelity prediction and privacy-preserving method is proposed based on the observable assembly deviations. A hierarchical graph attention network (HGAT) is established to predict the assembly feature deviations. The hierarchical generalized representation and differential privacy reconstruction techniques are also introduced to generate the graph attention network model for assembly deviation privacy-preserving. A derivation gradient matrix is established to calculate the defined modified necessary index of assembly parts. Two privacy-preserving strategies are designed to protect the assembly privacy of node representation and adjacent relationship. The effectiveness and superiority of the proposed method are demonstrated by a case study with a four-column hydraulic press.

## Introduction

The PLM (Product-Lifecycle-Management)-oriented value chain collaboration^1,2^ has become the latest way of improving competitiveness in the economy globalization. Intelligent diagnosis and maintenance of mechanical products is one of the most important part. Hydraulic equipment plays a significant role in the manufacturing industry^3^. After a long-time usage, the deviations of each part can be very different from the theoretical values due to the deformation away from ideal position^4^. The existing maintenance methods are almost blind, time-consuming, and laborious. Because the measurable deviations are limited in the complex assemblies. Complete deviations provide a wealth of guidance information for the assembly maintenance. Hence, the graph models^5^, a new branch of machine learning method, are proposed to predict the unknown deviations based on the feature graph^6,7^. Here, this research focuses on the privacy-preserving for the assembly deviation prediction. Data privacy^8^ is vital, because the deviations are sensitive and it is necessary to avoid the equipment information leakage derived from the graph models. The existing research focus on deviation allocation in the product design^9–11^ and assembly stage^12–14^. For example, Stefan et al.^15^ proposed a method for tolerance evaluation in product conceptual design stage. It allows designers to evaluate tolerances before the final geometry is defined. Zhou et al.^16^ proposed an assembly sequence deviation propagation model based on the assembly-feature adjacency matrix and geometrical-feature tolerance matrix. The influence of cumulative deviations of different assembly sequences on product assembly quality can be accurately and effectively evaluated. Besides, Liu et al.^17^ proposed a fluctuation evaluation and identification method based on a machining error propagation network. The sources of fluctuations in the machining process of the workpiece can be identified. However, the existing deviation prediction methods are mainly used for assembly quality control. Most studies focus on the design stage, and not consider the geometrical feature deviations during usage. Disregarding deformations, these studies fall within the category of rigid body assembly. Besides, the deviations generated in the service stage are not used to guide the product maintenance or improve the tolerance allocation^18^.

Furthermore, a mechanical assembly can be regard as a feature graph^19–21^. As the development of artificial intelligence, there is a promising branch to generalize the machine learning algorithms^22,23^ to graph domain^24–26^. And the missing deviations in the assembly are expected to be predicted based on its feature graph. In this paper, a hierarchical graph attention network (HGAT)^27–29^ was proposed to predict the unknown assembly deviations of the hydraulic equipment, and a derivation gradient matrix is defined for equipment maintenance. On the one hand, the hierarchical mechanism of proposed HGAT method is beneficial for utilizating graph structure information. On the other hand, the weights of adjacent nodes further improve the accuracy of the deviation prediction.

**Figure 1:**
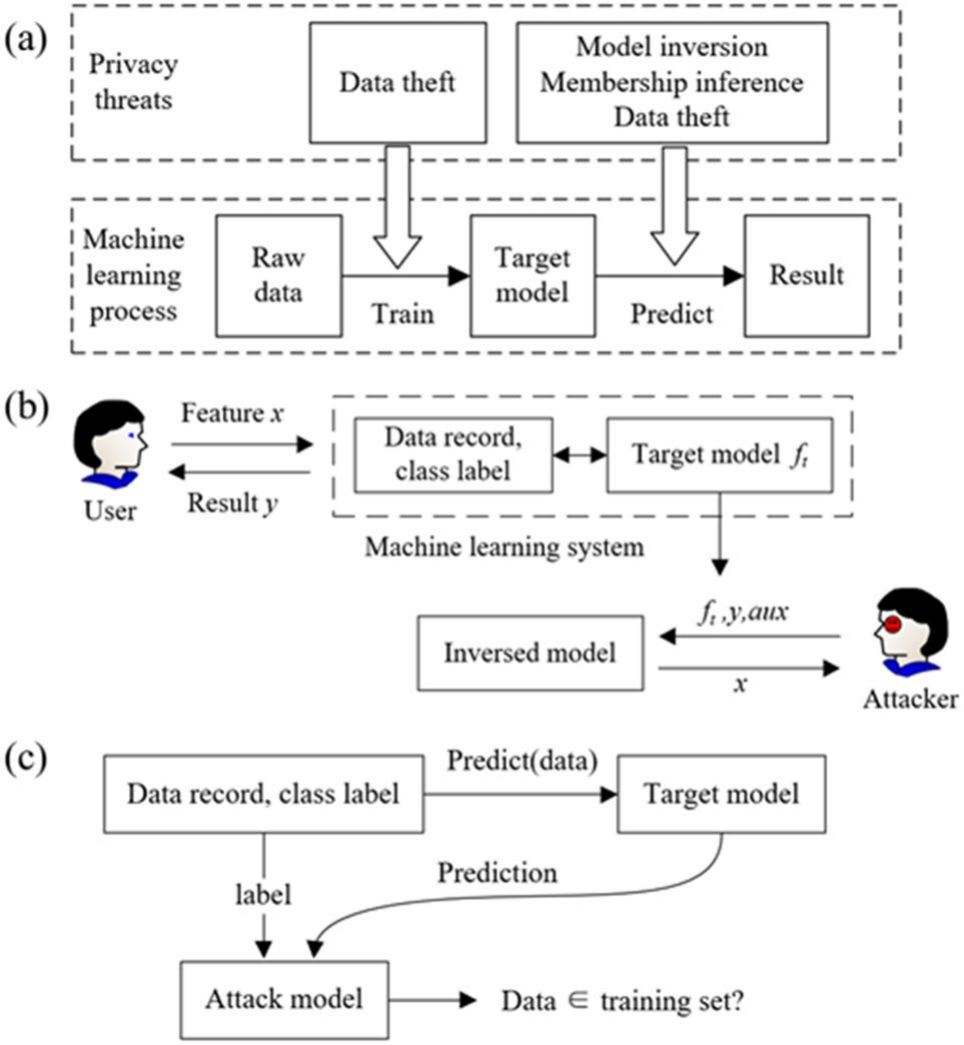
(**a**) Major privacy threats in the machine learning process, (**b**) the model inversion in privacy threats, (**c**) the membership inference in privacy threats.

The deviation prediction process contains two stages. The first is the training stage, where the training data are imported into the algorithm. To reduce the loss on the validation set, the training parameters and hyperparameters are optimized based on backpropagation. The second stage is prediction stage, using the target model to predict the deviations on the test set. Therefore, the potential privacy threats could happen in these two stages^30^ as shown in Fig. 1a. Specifically, during the training stage, it mainly faces the threat of data theft. During the prediction stage, there may be more threats such as model inversion, membership inference, and data theft. A high-performance model depends on both rich features and complete edge information in the graph. Surprisingly, there is very little research on privacy protection of high-performance graph neural network models^31–33^.

The data theft means the raw data have the risk of being stolen. Especially, in the application of a large-scale machine learning algorithm, it is necessary to train the algorithm on the server by the cooperation of many users. The cloud transmission of data increases the possibility of being attacked. Given that the attacker has some access to the target model, model inversion techniques aim to infer the class representation, as shown in Fig. 1b. ^34^. The attacker needs to provide some auxiliary, which could be some experience or human knowledge. The concept of model inversion is introduced by Fredrikson et al.^35^. They showed how the adversary using the outputs from a classifier to infer the sensitive features used as inputs. These techniques are sometimes described as violating the privacy of the training data, even though the inferred features are characterized by the entire class. The membership inference attack means infer whether a given data point belongs to the training dataset as shown in Fig. 1c. For example, if the heath records are used to train a classifier, discovering a specific record that was used to train will cause sensitive information leak of the individual.

In the present work, firstly, the graph model used to predict the deviations is proposed. The nodes the edges in the graph are defined according to the assembly relationship. Secondly, two privacy-preserving strategies are designed to protect the privacy of node representation and relationship in the graph. Compared with the related works in recent years, it has three contributions.

1. The hierarchical graph attention network (HGAT) is established to predict the unknown deviations of the assembly.
2. The derivation gradient matrix is processed based on the completed deviation data to calculate the defined modified necessary index (MNI).
3. A hierarchical generalized representation and a differential privacy reconstruction are designed to protect the node representation and adjacent matrix, respectively.

The following sections are organized as follows. The graph model used in the graph neural network is given in “Graph model establishment”. The methodologies employed for deviations prediction and privacies protection are described in “Methodology”. To verify the extensiveness and effectiveness of the proposed method a case study is discussed in “Case study”. The conclusion is summarized in “Conclusion”.

## Graph model establishment

**Figure 2:**
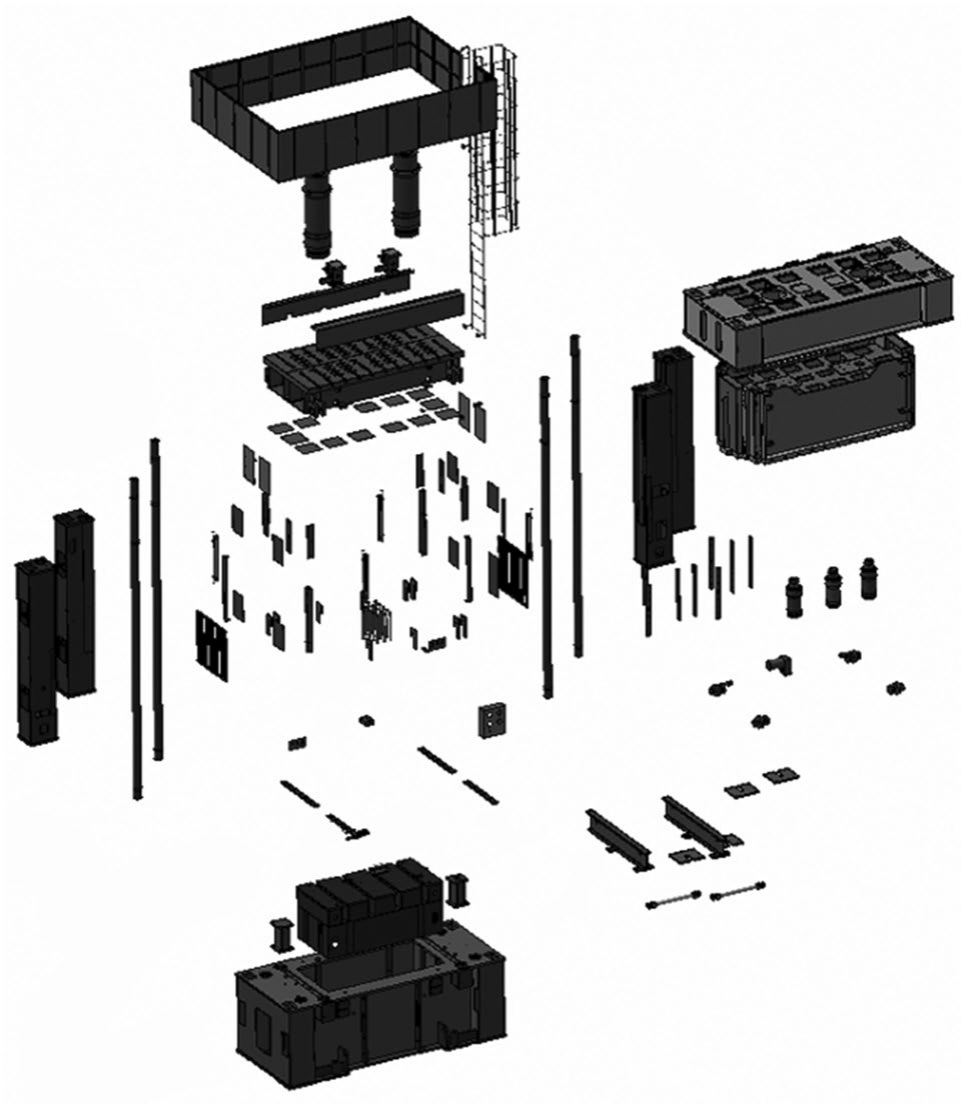
The exploded view of a hydraulic equipment.

A complex hydraulic equipment^36^ is composed of many parts as shown in Fig. 2. In fact, the assembly relationships can be represented by a feature graph. The definition of nodes and edges in the graph are introduced in this section. The establishment of the graph model is the basis of the graph neural network algorithm for the deviation prediction.

### The node definition

**Figure 3:**
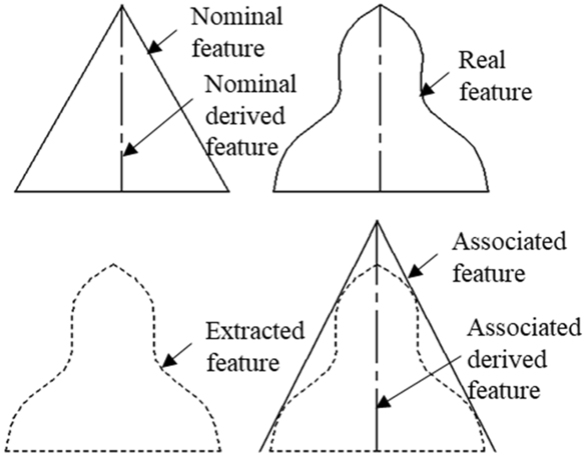
Four different features of a conical surface.

The unspoken details of the part features need to be clarified before defining the nodes. Taking the conical surface^37^ as an example, as shown in Fig. 3. The nominal feature is the conical surface on the nominal geometric model. Its axis is the nominal derivative feature. The so-called deviation means that the axis is derived from the conical surface. The real surface corresponding to the nominal feature obtained from the actual processing is the real feature. However, in the actual inspection process, only a limited number of these features were sampled. These are called extracted features. Then the ideal surface matched to the extracted point is called the associative feature. The derived elements of the association are derived from the associated elements. We define each node has up to two circles. The solid circle represents the real feature, and the associated derived feature is expressed as a dashed circle around the solid circle.

The node representation is used to train the network, which should contain as much information of the node as possible. Here we define the node representation *n* as:

1 $$ n = [x,y,z,\alpha ,\beta ,\gamma ,x,y,z,m]^{{\text{T}}} h = [u,v,w,\alpha ,\beta ,\gamma ,x,y,z,n]^{T} $$

where (*u*, *v*, *w*, *α*, *β*, *γ*) are the measured deviations that along and around the global coordinate system. (*x, y*, *z*) is the coordinate point of the local feature. *m* is the number of nodes in the shortest path between the part feature and the reference feature.

The node label is defined by a comprehensive indicator *l*_*c*_. It is defined as:

2 $$ l_{c} = \sqrt {w_{1} x^{2} + w_{2} y^{2} + w_{3} z^{2} + w_{4} \alpha^{2} + w_{5} \beta^{2} + w_{6} \gamma^{2} } $$

where *w*_*1*_, *w*_*2*_, *w*_*3*_, *w*_*4*_, *w*_*5*_, and *w*_*6*_ $({\mathrm{p}}_{1},{\mathrm{p}}_{2},{\mathrm{p}}_{3},{\mathrm{p}}_{4},{\mathrm{p}}_{5},{\mathrm{p}}_{6})$ are the weights corresponding to each element. Then the node label is determined by discretizing the value of *l*_*c*_.

### The edge definition

**Figure 4:**
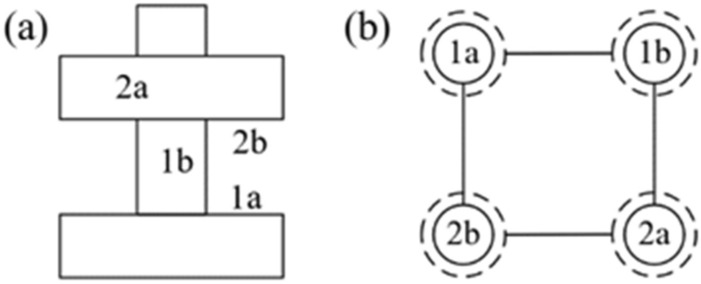
Toy example of feature graph in one component.

The edge is determined by the tolerance mode. Specifically, the self-reference tolerance is the edge that links the solid circle and the dashed circle of the same node. The cross-reference tolerance is the edge that links two dashed circles that corresponding to the same part. The fit tolerance is the edge that links two solid circles that corresponding to different parts. For example, the feature graph of one component is showed in Fig. 4. The assembly consists of two parts, parts 1 and 2. The surface of 1a has a perpendicularity tolerance with 1b, 1b has a fit tolerance with 2a, 2a and 2b have a perpendicularity relationship. Finally, there is a positioning relationship between 2b and 1a. The final feature graph is shown in Fig. 4b.

## Methodology

### Deviation prediction

**Figure 5:**
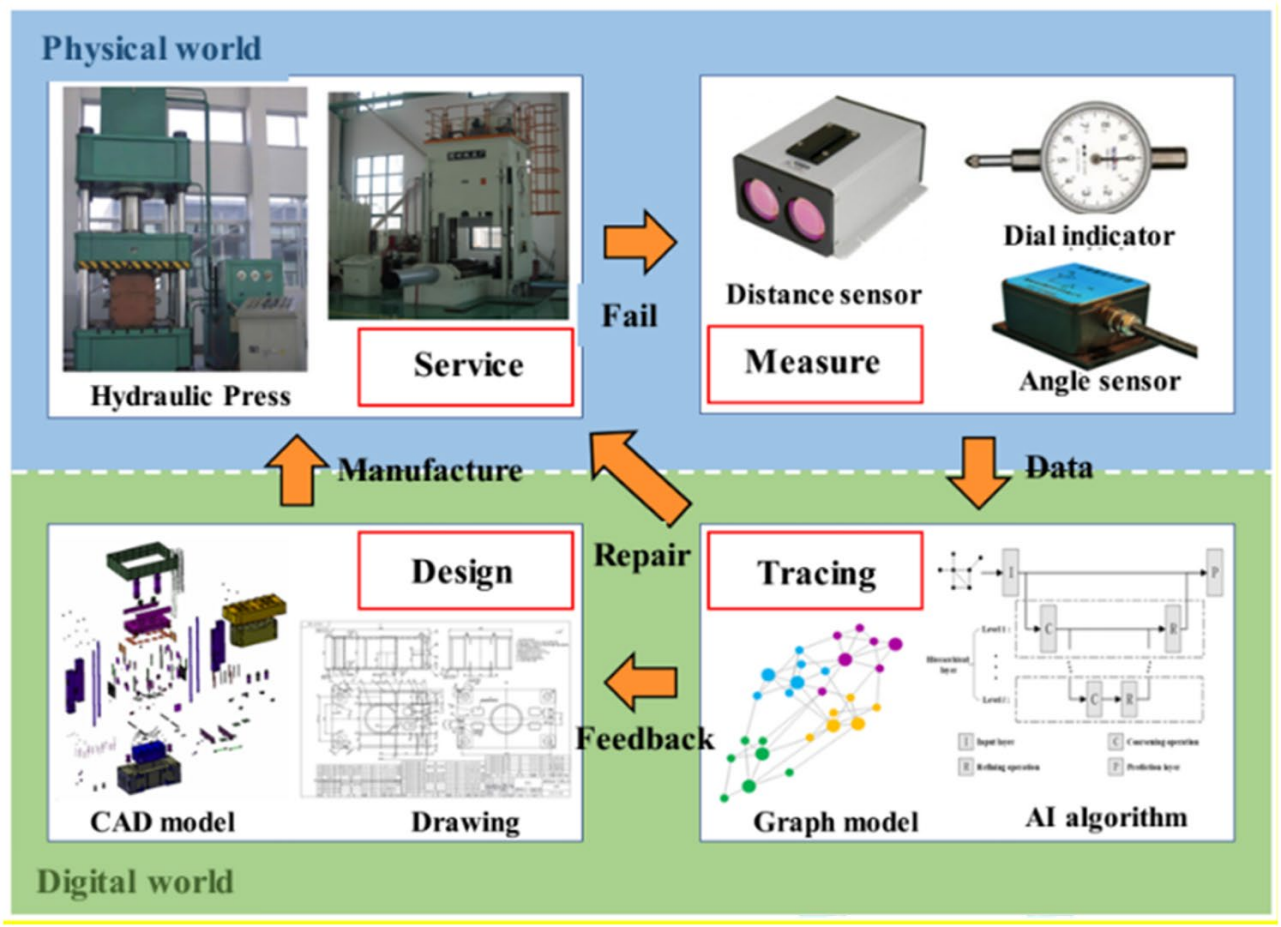
The framework of deviation prediction.

This section describes assembly deviation prediction and identifies the feature surfaces that have the greatest impact on the functional requirements of in-service complex mechanical assemblies. The assembly deviation prediction focuses on the difference between the surface of the part in service and its ideal position. Large discrepancies lead to assembly failures. The PLM-oriented value chain collaboration aims to improve the enterprise's core competitiveness, which combined the manufacturing system data with artificial intelligence, big data and other technologies to make the best decisions. This paper focuses on the collection and processing of product data, manufacturing data and process data in the manufacturing systems. Based on the background of PLM-oriented value chain collaboration, a framework of deviation prediction for complex mechanical assemblies is presented. As shown in Fig. 5, a closed-loop feedback of measured deviations is introduced to the design framework, which helps to extend the service life of the products.

The process of the assembly deviation prediction includes four stages. The first stage is the product design stage. The design engineers build a three-dimensional model of the assembly and make detailed two-dimensional drawings based on design experience, including the overall dimensions of each part and detailed tolerances. The second stage is the product service stage. Workers in the manufacturing department process mechanical products according to the requirements of the drawings. After the quality inspection, the assembled products can be put into service. The third stage is the deviation perception stage. When the assembly functions no longer meet the design requirements, the deviation information collected by each sensor is used to prepare for product maintenance and product upgrade. The fourth stage is deviation tracing. Based on a small sample of measured deviations, all the unmeasured deviations are calculated by the machine learning method. The deviations are traced through the deviation gradient matrix. And further judge whether the deviations are caused by the structure deformation or the unreasonable tolerance design. If it is caused by the structure deformation, repair or replace the product. If it is caused by an unreasonable tolerance design, a tolerance reallocation design is necessary. Deviation tracing in this study aims to find unreasonable tolerance allocations in the design process or feature surfaces that no longer satisfy the precision requirements due to product service. This research only focuses on the situation in which the actual position of the surface deviates from the ideal position. Define the necessary modify index MNI_*ij*_ based on the node label *l* as

3 $$ {\text{MNI}}_{ij} = \left| {l_{i} - l_{j} } \right| $$

where *i* and *j* are the node subscripts.

In the process of the deviation prediction, a certain amount of data is input to support the training of neural network. Generally, the more the number of markers, the higher the accuracy of the prediction. However, if there is insufficient deviation data for testing, all the input deviation data generated by the simulation can be used to debug the hyperparameters of the HGAT model. This study assumes that the deviations obey a normal distribution. Once the interval of FRs is known, the mean and the variance values of deviations can be calculated using the 3σ criterion^38^. Then the unknown labels can be obtained by importing the measured data into the HGAT model. We can take the value if there is more than one set of test data. After all the node labels are obtained, it is important to identify the tolerance values that need to be redesigned. The recognition process is based on the value of MNI_*ij*_. The larger the MNI_*ij*_ value, the more likely the error source is. And a higher accuracy of this tolerance should be prioritized to avoid the deviation transmission. The accuracy grade of adjustment is related to the processing cost, which is not considered in this research. Replace the corresponding elements in the adjacency matrix with MNI_*ij*_ to obtain the deviation gradient matrix *S*. The calculation of *S* can be carried out by the following formula:

4 $$ S = {\text{abs}}\left( {A*D - D^{T} *A^{T} } \right) $$

where abs() is the absolute value function, * is the dot product operation, *A* is the adjacency matrix of the feature graph, and *D* is the deviation vector composed of all nodes in the feature graph. It is a node label composed of one-dimensional vector. It should be pointed out that *S* can only help engineers propose strategies for tolerance improvement, it cannot directly indicate what specific value should be increased.

The proposed HGAT framework can be divided into three parts: input layer, hierarchical layer and prediction layer. A GAT algorithm was used in the input layer and a multi-head mechanism was used to stabilize the learning process. The input of the input layer is the initial graph N0 and the output node representation *N*_1_ can be calculated as follows^39^:

5 $$ N_{1} { = }\bigcup\limits_{i = 1}^{I} {\sigma \left( {\alpha^{i} T^{i} N_{0} } \right)} $$

where $I$ is the number of head, $T^{i}$ is the transformation matrix, $\sigma \left( {} \right)$ is the nonlinear ELU activation function, and $\alpha^{i}$ is the regularized graph attention coefficient matrix.

There are* l* layers in the hierarchical layer. Each layer consists of two types of symmetrical operations, called coarse calculation and refined calculation. Inspired by k-way partitioning scheme^40^, the connection strength between node $v_{j}$ and node $v_{k}$ is defined as:

6 $$ s_{i} \left( {v_{j} ,v_{k} } \right) = \frac{{A_{i} \left( {v_{j} ,v_{k} } \right)}}{{\sqrt {D_{i} \left( {v_{j} } \right)D_{i} \left( {v_{k} } \right)} }} $$

where $A_{i}$ and $D_{i}$ are the adjacency matrix and the degree matrix of graph $G_{i}$.

The coarse calculation of the graph is a kind of contraction operation which captures global structure and ignores details. Based on the contraction set, the contraction matrix $M_{i} \left( {r,h} \right)$ is defined as

7 $$ M_{i} \left( {r,h} \right) = \left\{ {\begin{array}{*{20}c} {\frac{1}{{|V_{i}^{r} |}},{\text{if }}v_{h} \in V_{i}^{r} ;} \\ {0,{\text{otherwise}}.} \\ \end{array} } \right. $$

where $V_{i}^{r}$ denotes the contraction set of graph $G_{i}$. Thus, the node representation and the adjacency matrix of graph $G_{i + 1}$ can be calculated as follows;

8 $$ \begin{gathered} N_{i + 1} = M_{i} N_{i} \hfill \\ A_{i + 1} = M_{i} A_{i} M_{i}^{T} \hfill \\ \end{gathered} $$

The refined calculation is introduced to restore the graph structure reduced by the coarse calculation. Based on the refined calculation, the node representation is defined as

9 $$ N_{i + 1} = M_{2l + 1 - i}^{T} N_{i} $$

The detailed process of hierarchical layer calculation is described as follows.
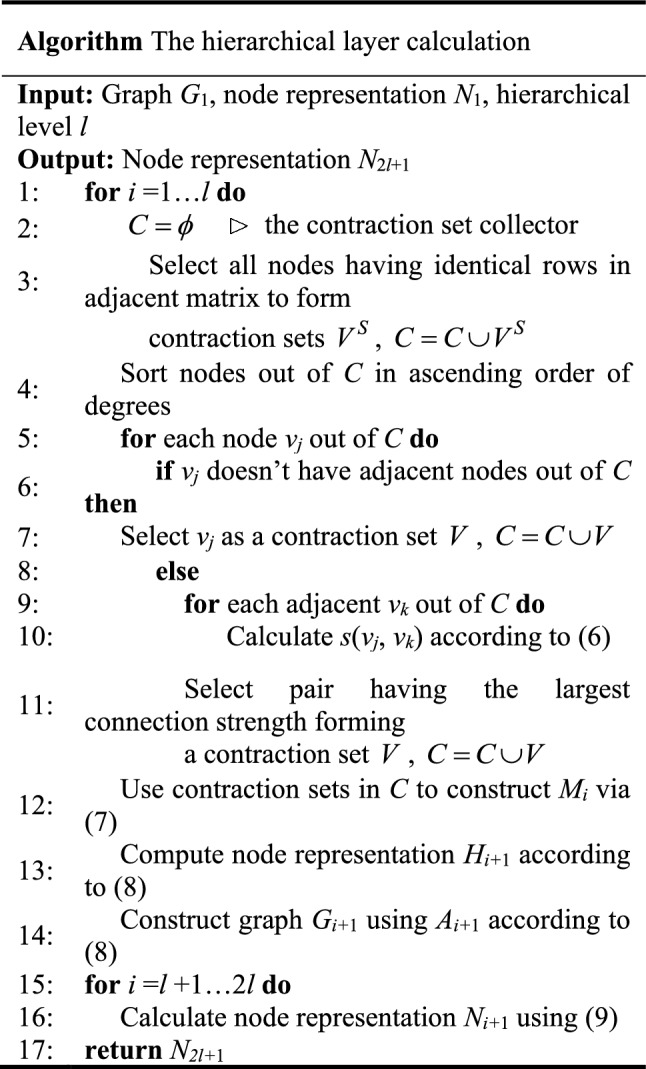


At last, a softmax classifier is added in prediction layer to predict the one-hot encoded node labels, which reflecting the magnitude of the deviation.

**Figure 6:**
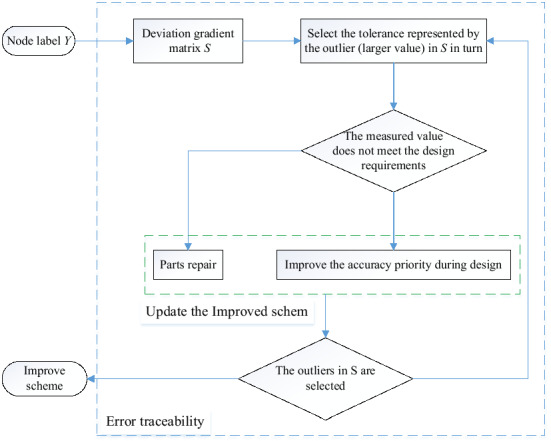
Error traceability process.

The error traceability process is shown in Fig. 6. According to the node labels, the tolerance represented by the outliers in *S* is selected for the actual test. When the design requirements are not met, it means that the tolerance constraints are no longer satisfied due to wear or force. The corresponding parts should be processed again or replaced directly. If it is within the design range, the accuracy grade of the design scheme needs to be improved. Finally, output the improvement scheme.

### Privacy-preserving

The purpose of this section is to increase the privacy-preserving ability of the graph neural network algorithm. More specifically, we focus on the preservation of node representation and relationships in the graph.

#### A The node representation protection 

**Figure 7:**
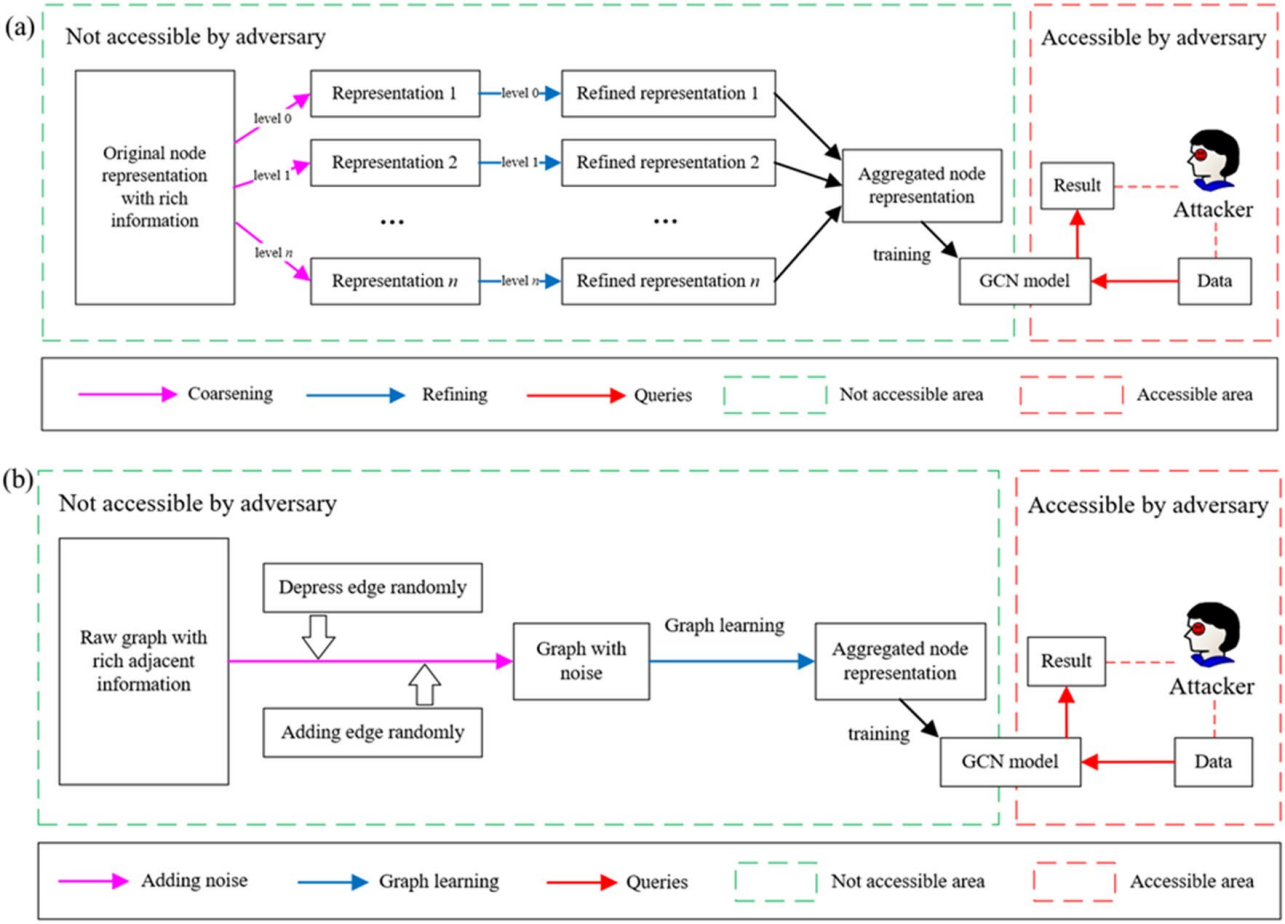
Overall of preserving approach: (**a**) node representation, (**b**) adjacent matrix.

To protect the node representation, a hierarchical generalized representation is proposed as shown in Fig. 7a We train the graph neural network model in the local server. Thus, it is not accessible by the adversary. In the application of the HGAT model, the node representation faces a hazard of being attacked. We employ the coarsening procedure to calculate the node representation in different coarsening levels. Then we refine the graph to have the representation of each original node. Afterward, we use the aggregation method to synthesis new node representation. Thus, the new node representation contains classified information of the original one, even if it is attacked, it will not leak the node privacy directly.

#### The adjacent matrix protection 

To protect the adjacent matrix, a differential privacy reconstruction is proposed as shown in Fig. 7b Similarly, we classify the process accessible by the adversary (red dotted box in Fig. 7b) and not accessible by the adversary (green dotted box in Fig. 7b). We start with the raw graph with rich adjacent information. Noises are added by depressing and adding the edges randomly. To reduce its negative effects on the subsequent training process, a graph learning layer is added to remodify its adjacent matrix. Then the node representation is calculated according to the adjacent matrix. Therefore, the raw graph is protected by the proposed methodology.

## Case study

**Figure 8:**
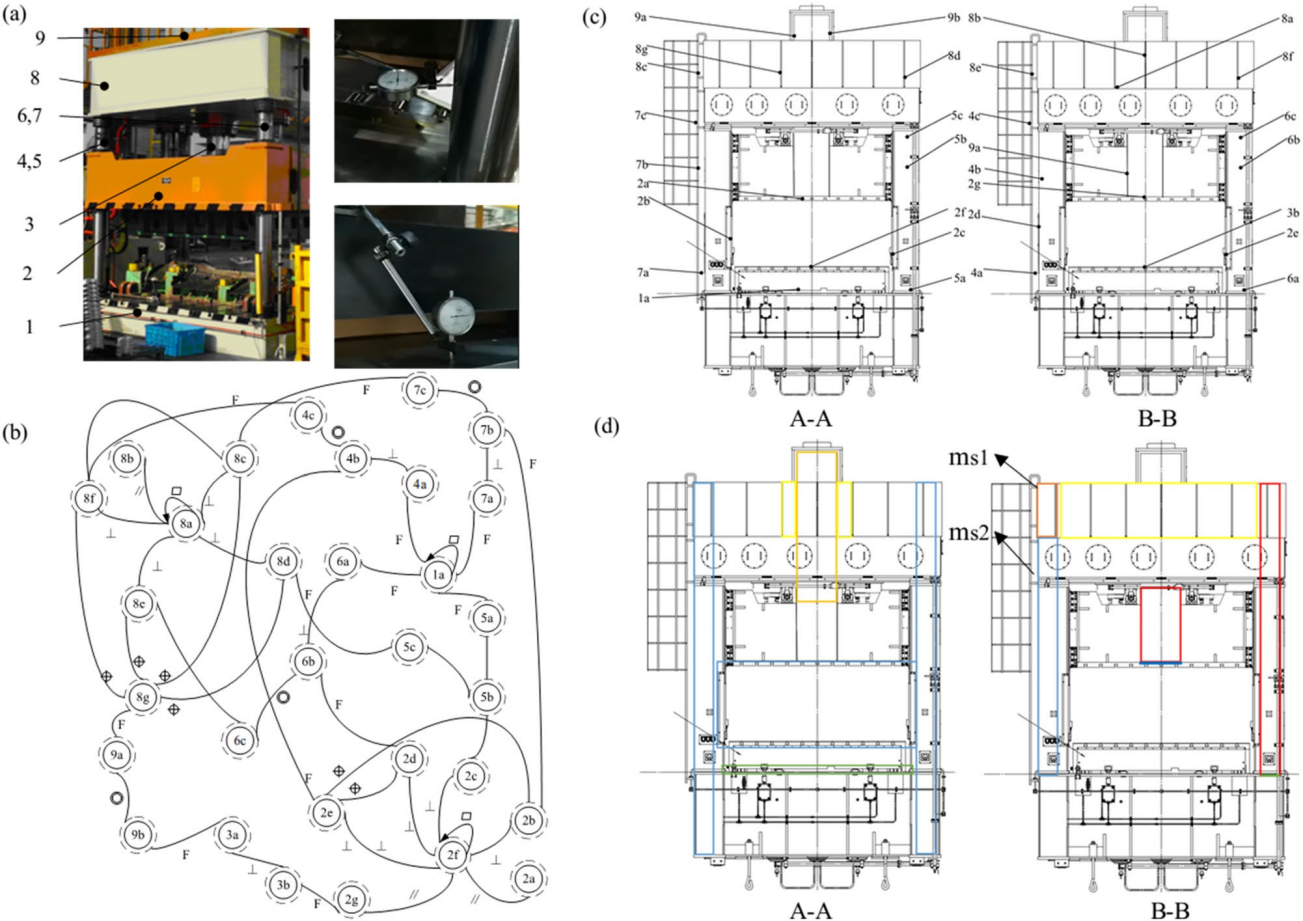
Case study: (**a**) Schematic diagram of the four-column hydraulic press to be repaired and the deviation collection on site, (**b**) feature graph of four-column hydraulic equipment, (**c**) simplified three-dimensional hydraulic equipment assembly and node definition, (**d**) all node labels of four-column hydraulic press represented in 5 colors.

The four-column hydraulic equipment shown in Fig. 8a consists of many parts. But not every part is essential for feature graph molding. To simplify, the key parts of hydraulic equipment have been selected, such as the worktable (1), the slider (2), the piston rod (3), the columns (4, 5, 6 and 7), the beam (8), and the cylinder (9). Due to the frequent reciprocating movement of the slider, the accumulated deviation is the largest when the piston rod reaches the maximum position and the slider moves to the lowest point at the same time. Therefore, this study chooses this position to establish the feature graph. The simplified three-dimensional hydraulic equipment assembly and detailed definition of the nodes are shown in Fig. 8c. The feature graph of four-column hydraulic equipment is shown in Fig. 8b. There are 31 nodes and 47 edges in the feature graph, and the adjacency relationship of nodes are shown in Table 1.

**Table 1:** Adjacency relationship of nodes in the feature graph.

Node	Adjacent node	Node	Adjacent node	Node	Adjacent node	Node	Adjacent node
1a	1a,7a,5a,4a,6a	3a	9b,2b	6a	1a,6b	8c	7c,8a,8g
2a	2f	3b	2g,2a	6b	6a,6c,2d	8d	5c,8a,8g
2b	7b,2c,2e,2f	4a	1a,4b	6c	6b,8e	8e	6c,8a,8g
2c	5b,2b,2d,2f	4b	4a,4c,2e	7a	1a,7b	8f	4c,8a,8g
2d	6b,2c,2e,2f	4c	4b,8f	7b	7a,7c,2b	8g	8c,8d,8e,8f,9a
2e	4b,2b,2d,2f	5a	1a,5b	7c	7b,8c	9a	8g,9b
2f	2a,2b,2c,2d,2e,2f,2g	5b	5a,5c,2c	8a	8a,8b,8c,8d,8e,8f	9b	9a,3a
2g	2f,3b	5c	5b,8d	8b	8a		

The 31 nodes in the feature graph are divided into three types of data sets, as shown in Table 2. The ratio of the test set to the verification set is the same as that of the standard data set. The training set is used to train the neural network, which including all the nodes with labels. The validation set is used to select hyperparameters. The test set is used to test the accuracy of the algorithm. The labels of all the nodes are used to debug the proposed model.

**Table 2:** Date sets.

Training set	Validation set	Test set
1a, 2a, 2f, 3a, 4b, 5b, 6b, 7b, 8a,8b	2b, 2d, 2g, 3b, 4a, 5a, 6a,7a,8d,8f, 9a,	2c, 2e, 4c, 5c, 6c,7c,8c, 8e,8g, 9b

Next, calculate the deviation gradient matrix based on the test data. Based on the simulation data, the main hyperparameters obtained by debugging are as follows: the classification level is 1, the weight decay is set to be 0.01, and there is no dropout. The input layer contains 8 attention headers. The deviations are shown in Table 3. The labels are defined according to their *l*_*c*_ values. The on-site measurement process of physical objects and deviations of the hydraulic press to be repaired are shown in Fig. 8a. Limited by the conditions, a micrometer tool with a special measuring tool are used for measurement. The inverse trigonometric function formula is used to convert the measured distance data into angle data. For the feature surfaces that are difficult to test, the deviations are assumed to obey a normal distribution in the FR interval, and the deviation data are generated by the Monte Carlo simulation method.

**Table 3:** Deviation data of hydraulic equipment.

Node	Rotation angle around x axis (°)	Rotation angle around y axis (°)	*l*c	label
1a	− 2.0e−5	0.0e−5	0.003	1
2a	3.3e−4	6.8e−4	0.200	2
2f	− 1.1e−4	5.3e−4	0.153	2
3a	− 2.6e−3	1.2e−3	0.498	5
4b	1.6e−4	− 5.3e−4	0.154	2
5b	3.7e−4	6.7e−4	0.198	2
6b	3.0e−5	1.5e−3	0.421	5
7b	− 8.0e−5	5.2e−4	0.148	2
8a	3.9e−4	7.5e−4	0.221	3
8b	2.1e−4	− 7.2e−4	0.209	3

The nodes 1a-8b in Table 3 represent the upper surface of the worktable (1), the upper surface of the slider (2), the lower surface of the slider (2), the outer surface of the piston rod (3), the outer cylindrical surface of the column (4), the outer cylindrical surface of the column (5), the outer cylindrical surface of the column (6), the outer cylindrical surface of the column (7), the lower surface of the upper beam (8), and the upper surface of the upper beam (8), respectively. Before calculating the deviation gradient matrix *S*, the HGAT model is used to predict the missing labels by minimizing the cross-entropy loss of the data in Table 3. As shown in Fig. 8d, a color map (Table 4) is used on the graph to represent all node labels. These predicted values can provide a reference for maintenance. The slider (2) and one side of the piston rod (3) connected to the slider are deformed greatly due to the deformation of the column (6). The deviation of the connection side between the upper beam (8) and the column (4) is large. Therefore, according to actual production experience, replacing the column (6) and adjusting the upper and lower bolts m_s1_ and m_s2_ to make the upper beam level is the best maintenance strategy.

**Table 4:** Node label category and corresponding description.

label	1	2	3	4	5
*y*_*c*_	yc < 0.107	0.107 ≤ yc < 0.203	0.203 ≤ yc < 0.299	0.299 ≤ yc < 0.395	yc ≥ 0.395
Degree	Slightly	Little	Medium	Very	Serious
Color					

The tolerance values that need to be improved are determined according to the deviation gradient matrix *S*. The values of *S* are shown in Table 5, and the values not listed in the table are all 0. The first four maximums in *S* represent edges 6a–6b, 2c–5d, 2d–5e, and 2d–5f, respectively. The values are 4, 3, 3, and 3 respectively. It is assumed that the actual tolerances are still within the design range at this time. To improve the tolerances, the verticality of the outer surface and the step of the column, the position tolerance of the four holes on the slider, and the verticality of the four holes on the slider to its lower surface should give a higher accuracy value.

**Table 5:** Deviation gradient matrix value.

Row	Column	Value	Row	Column	Value	Row	Column	Value
3a	9b	1	5e	2d	3	8d	8a	1
3b	2 g	1	5f.	2d	3	8e	8a	1
4b	4a	1	6b	6a	4	8 g	8d	1
4c	4b	1	7b	7a	1	8 g	8e	1
5b	5a	1	8c	7c	1	9a	8 g	1
5d	2c	3	8e	6c	1			

## Conclusion

Machine learning models provide new possibilities to gain high-fidelity prediction based on existing observable assembly deviations during usage. These deviations are useful for product redesigns and repairs throughout the product life cycle. In this paper, a novel privacy-preserving method for assembly deviation prediction was proposed based on the HGAT algorithm. The HGAT is established based on the defined feature graph to predict the unknown deviations of the assembly. Afterwards, to-be-improved deviations are identified based on the deviation gradient matrix. Then, two strategies are introduced to protect the privacy of deviations. The hierarchical generalized representation and the differential privacy reconstruction are constructed based on the HGAT algorithm to protect the privacy of node representation and the adjacent matrix, respectively. Finally, a four-column hydraulic press is selected to verify the feasibility and superiority of the proposed method. The proposed method is effective and has the advantage of predicting assembly deviations caused by unknown deformation derivations during usage. The prediction accuracy of assembly deviations is guaranteed by assigning different weights to adjacent nods, while the HGAT guarantees the efficiency of the whole method. Also, it can be widely used to handle the other data issues under PLM-oriented value chain collaboration. Such as visual relationship detection and stock movement prediction.

However, there are also some limitations and disadvantages. The node representation is relatively simple that only consists of tolerance chain information and node location information. The proposed method is based on some assumptions that may not be satisfied in the actual situation. For example, the derivations obey a normal distribution. The priority of accuracy improvement is proposed, but the quantitative analysis is missing.

Thus, the future directions can be concluded as follows. The machine learning model is in development and may lead to an improved node representation with sufficient graph information. The qualitative analysis is limited in deviation degree as well as maintenance location, suggesting quantitative analysis to determine the critical values of to-be-improved deviations. With respect to tolerance allocation, the multi-objective optimization model should be established with the consideration of actual manufacturing cost for different accuracy levers.

## Data Availability

The datasets used and/or analyzed during the current study are available from the corresponding author on reasonable request.
